# Reconsidering the Selection Strategy in a Flemish Honey Bee Breeding Program: Towards Selection by Exclusion

**DOI:** 10.3390/insects17070689

**Published:** 2026-07-02

**Authors:** Emma Bossuyt, Ellen Danneels, Dirk C. de Graaf

**Affiliations:** 1 Honeybee Valley, Department of Biochemistry and Microbiology, Faculty of Sciences, Ghent University, Krijgslaan 287, S33, 9000 Ghent, Belgium; ellen.danneels@ugent.be (E.D.); dirk.degraaf@ugent.be (D.C.d.G.); 2 Laboratory of Molecular Entomology and Honeybee Pathology (L-MEB), Department of Biochemistry and Microbiology, Faculty of Sciences, Ghent University, Krijgslaan 287, S2, 9000 Ghent, Belgium

**Keywords:** honey bee, mass selection, breeding, Varroa resistance, selection by exclusion

## Abstract

Selective breeding is widely used to improve honey bee health and colony performance, commonly applying selection based on colony traits. Since 2017, a mass selection program in Flanders, Belgium, has ranked queens according to resilience, productivity and behavior traits. Using longitudinal data (2017–2024), we evaluated the effectiveness of this strategy by assessing colony performance across years and generations. Behavioral traits showed the clearest improvement over time, whereas progress in productivity and especially resilience-related traits remained limited. Offspring of top-ranking queens frequently failed to achieve overall ranking values comparable to those of their mothers; rather, they scored lower. For the traits Varroa index and spring development, offspring from lower-scoring queens often performed better, while offspring from higher-scoring queens tended to perform worse. These findings indicate that the current mass selection approach did not consistently result in cumulative genetic improvement. Factors such as open participation, limited breeding standardization, and insufficient paternal genetic control may have reduced selection efficiency. These results highlight the opportunities and challenges of implementing an accessible honey bee breeding program. Refinement of the current selection framework and evaluation of alternative strategies, such as selection by exclusion, may therefore be necessary to achieve more robust and resilient honey bee populations.

## Simple Summary

Honey bee breeding programs are increasingly used to improve colony health, productivity, and resistance against parasites such as *Varroa destructor*. Since 2017, a large-scale breeding program in Flanders, Belgium, has selected queens based on behavioral, productivity, and resilience-related traits. In this study, we evaluated the effectiveness of the mass selection strategy using data collected from 2017 to 2024. Behavioral traits showed the greatest improvement over time, whereas progress in productivity- and resistance-related traits remained limited. In addition, daughter queens from top-performing mothers often did not achieve similar performance levels and frequently scored lower than their mothers. These findings suggest that the current mass selection strategy has limitations in achieving long-term genetic improvement. Factors such as the program’s open structure, limited standardization, and uncontrolled mating likely reduced selection efficiency. Our results highlight both the opportunities and challenges of the current honey bee breeding program and indicate that adjustments may be necessary to improve long-term colony performance.

## 1. Introduction

Selective breeding in apiculture has been practiced for several decades. The initial focus was to intensively breed bees with high honey production and gentleness [1]. However, the introduction of the parasitic mite *Varroa destructor* in Europe has fundamentally shifted breeding priorities in beekeeping. This ectoparasite severely affects honey bees by reducing flight performance, decreasing life expectancy and vectoring viruses [2,3,4]. Initial responses relied heavily on chemical treatments (acaricides) to control Varroa infestations. However, this approach has proven unsustainable because of the development of acaricide resistance in mites and the accumulation of chemical residues in hive products [5,6]. Evidently, these limitations highlight the need for alternative sustainable solutions to manage Varroa plagues.

A promising approach is the natural development of resistance to Varroa in honey bees. Evidence from untreated colonies in France has demonstrated that some populations can survive long-term Varroa infestations without human intervention [7]. Similarly, the well-documented Gotland bee population, isolated colonies infested with Varroa on the Swedish island of Gotland, developed co-adaptation with *V. destructor* [8]. In these cases, natural selection has led to a stable host–parasite equilibrium without knowing the critical underlying mechanisms. This approach reduces the need for extensive Varroa resistance-related trait testing and thereby lowers the workload for beekeepers [9,10]. However, this strategy also has drawbacks, including high colony losses, reduced genetic diversity, increased aggressiveness, higher tendency for swarming, lower productivity, and practical challenges in maintaining isolated populations under consistent selection pressure [7,8,9,11].

An alternative, less risky strategy is human-mediated selection, which focuses on enhancing traits associated with Varroa resistance, such as grooming, Varroa sensitive hygiene (VSH), or mite non-reproduction (MNR). A well-known example at the USDA-ARS Honey Bee Breeding, Genetics and Physiology Laboratory is the Russian Honey Bee (RHB) program, which developed a novel honey bee stock derived from Varroa-resistant populations in far eastern Russia [12]. Comparative studies have shown that RHB colonies exhibit reduced brood attractiveness, increased brood removal, and enhanced grooming behavior compared to Italian honey bee colonies [13]. In addition to selection programs focused solely on resistance traits, more comprehensive selection approaches aim to enhance multiple traits simultaneously, balancing Varroa resistance with productivity and behavior. This is the case for the German breeding program AGT (“Arbeitsgemeinschaft Toleranzzucht”), which employs a rigorous and systematic approach by first pre-selecting colonies within a large population through annual trait testing based on the Ruttner (1972) protocol [5]. This testing process applies strict selection criteria to ensure the reliability and genetic diversity of selected stocks. For instance, a minimum of 20 bee colonies (from the same subspecies) must be tested, queens must be sourced from multiple beekeepers (not their own) to ensure objectivity, controlled mating is required and inbreeding is prevented [14]. Queens are compared using estimated breeding values, allowing breeders to predict the genetic potential of the queens and optimize mating decisions [5,15]. Second, the program will subject these pre-selected colonies to a survival test, defined as overwintering untreated colonies, while systematically monitoring the hives for mite infestation and bee population development [5]. Subsequently, colonies exhibiting high levels of Varroa resistance were selected for breeding.

The selective breeding program of Honeybee Valley (HBV, at Ghent University, Flanders, Belgium) also aims to improve colony resilience while maintaining productivity and ease of handling. This program, launched in 2016 with its first full testing season in 2017, formed the foundation of the dataset analyzed in this study. It is linked to existing European breeding efforts but adopts a more flexible approach than the latter. The HBV program allows the inclusion of colonies with diverse genetic backgrounds (or unknown), different honey bee subspecies, natural mating and beekeepers to test their own colonies. This program follows a mass selection protocol in which holistic traits are evaluated (i.e., Selection on Own Performance/Phenotype [16]). In contrast to the AGT program, the trait results are compared only with those of all participating colonies within the same year, rather than with outcomes from previous generations (i.e., breeding values). Each year, the colonies’ absolute trait results were ranked from best to worst and subsequently converted into relative values reflecting their position within the entire group of colonies for that year. Further breeding is recommended for a subset of queens, called top queens.

To evaluate the effectiveness of the mass selection strategy, this study addressed two overall questions. First, has mass selection led to measurable improvements in colony performance across years and generations? Mass selection assumes that consistently selecting high-performing queens will result in cumulative genetic gains and improved offspring performance. Therefore, we assessed both temporal trends in colony performance across testing years and generational trends between mother queens and their offspring. By analyzing longitudinal data gathered from 2017 to 2024, we assessed whether progress has been made in certain characteristics, particularly those related to resilience (against *V. destructor*), without compromising productivity or ease of handling. Second, irrespective of the outcome of the first question, we investigated whether the current selection strategy could be further optimized. Specifically, we evaluated whether the relatively flexible selection criteria applied within this program provide sufficient selection pressure to achieve genetic improvement or whether alternative approaches, such as more stringent selection or modified selection thresholds, are more effective. Together, these research questions aim to clarify the extent to which current mass selection practices translate into expected breeding gains and assess whether adjustments to the selection strategy are necessary to maximize the effectiveness of future honey bee breeding efforts.

## 2. Materials and Methods

### 2.1. Data Collection

Data was collected from 2017 to 2024, as part of the Flemish honey bee selection program at HBV. Participation in the selection program was voluntary, and beekeepers could enroll any year with varying numbers of colonies. These colonies came from different regions in Flanders, Belgium, which share broadly comparable climatic conditions. Information on the genetic and management background of the colonies was collected where available, but was not required for inclusion. No restrictions were imposed on the bee subspecies of the participating colonies. All traits were assessed from the autumn of the year before (first measurement of the winter index) until the summer of that testing year (last honey harvest). Beekeepers were instructed not to apply chemical treatments to their tested colonies during this period. No additional strict inclusion criteria were imposed; however, queen testing was conducted within the standardized framework of the selection program (Supplementary Materials, Figure S1). All data, including queen background information and test results, were centralized through our web-based application Breed It (https://www.honeybeevalley.eu/breedit, accessed on 2 February 2026). This background information included the lineage (maternal and paternal line) and the mating type (drone line) of the queen. Based on this information, and as queens were tested over multiple consecutive years, the dataset included related lineages. As the selection program recommended breeding from top-performing queens, the tested offspring descending from these queens were identified. Consequently, the analysis was restricted to offspring of top queens, because offspring of lower-performing queens were sparsely represented.

### 2.2. Test Protocols

The traits included in this breeding program were based on Uzunov et al. [17] and comprised behavioral traits (gentleness, calmness and swarming tendency), productivity traits (honey yield and spring development), and resilience traits (winter index, Varroa index, hygienic behavior, suppressed mite reproduction (SMR; later MNR and mean Varroa reproduction rate (mVR)) and “suppressed *in ovo* virus infection” (SOV) status). Behavioral traits were assessed following the scoring protocol described by Büchler et al. [18]. The description of productivity and resilience traits is based on Bouuaert et al. [19], with adjustments implemented in recent years as outlined below.

Honey yield was initially defined as the total amount of honey harvested per colony in a single season. From 2021 onwards, this measure was standardized at the apiary level and expressed as the relative contribution of a colony to the apiary average (i.e., honey index = (colony honey yield/apiary average honey yield) × 100) to account for environmental variation. Thus, colonies with values > 100 produced more honey than the apiary average, whereas colonies with values < 100 produced less. The spring development was calculated as the brood area (dm^2^) measured in April (just before super placement) divided by the brood area measured in February (first post-winter inspection), thereby representing the relative increase in brood area during spring.

In our selection program, hygienic behavior was assessed as the removal of dead (pin-killed) brood [18]. From 2017 to 2020, the fully removed cells were counted 24 h after perforation. In 2021, multiple assessment timings were used, while from 2022 onwards, evaluation was standardized to 8 h, as 24 h measurements consistently resulted in complete (100%) removal and thus lacked variation. Next, the Varroa index is a measure of Varroa mite population growth between early spring and summer, calculated as the number of phoretic mites collected by the sugar method on 30 g of nurse bees in July (= Varroa end-infection) divided by the average daily mite fall over three weeks during the first main spring pollen-producing bloom (February–March) (= Varroa begin-infection). Because sample weights were not always exactly 30 g, mite counts were standardized and expressed as the number of mites per 10 g of bees before calculation of the Varroa index. The winter index was determined as the ratio of colony size (number of occupied frames) in February relative to October.

Varroa reproduction was evaluated using (preferably) drone brood sampled in May–June at the purple-eyed pupal stage (17–18 days). The full phenotypical analysis protocol is mentioned in Appendix A. From this analysis, Varroa reproduction parameters could be determined. Initially, SMR (here referred to as Varroa non-reproduction index) was presented as the proportion of non-reproductive mites compared to all infested cells (multiple infested cells, MICs, and single infested cells, SICs). From 2022 onwards, this was replaced by two parameters: MNR, defined as the proportion of SICs without offspring over the total number of SICs, and mVR, defined as the average number of offspring per infested cell while compensating for the reduced number of offspring with increasing proportions of MICs [20]. A minimum of 10 (single) infested cells was required for reliable estimation.

The SOV trait was used as an exclusion criterion. In early spring, samples of 10 drone eggs were collected and screened for the presence of four viruses: Acute Bee Paralysis Virus (ABPV), Deformed Wing Virus (DWV) (all genotypes), Black Queen Cell Virus (BQCV) and Sacbrood Virus (SBV). Colonies were classified as SOV-positive (no viruses detected) or SOV-negative (≥1 virus detected). A detailed protocol is provided in de Graaf et al. [21] and Claeys Bouuaert et al. [22]. The evolution of this trait is not mentioned thoroughly here, as this trait was evaluated separately [23].

### 2.3. Ranking of Test Results

For each trait, test results were ranked from best to worst and divided into four quarters based on boxplot distributions: Q1, median and Q3. A “Q-value” was assigned to each trait result, corresponding to its quarter position (Table 1). These Q-values were then further used to derive a final global ranking of all queens.

The method for global ranking has changed over time (Table 2). From 2017 to 2022, the Q-values were first summed per different trait categories (behavior, productivity and resilience). In subsequent calculations, more weight was given to resilience traits (50%) than to behavior and productivity traits (25%). Overall results of these calculations were ranked and again divided into quarters based on boxplot distributions. Only the 50% best-scoring queens (queens in the first and second quarter), who were also SOV-positive, were labelled as top queens. From these queens, further breeding was recommended. From 2021 onwards, an additional criterion was introduced, requiring a minimum of three assessed resilience traits for a queen to be eligible for top queen classification, ensuring comparability between individuals with differing levels of phenotypic information. Since 2023, the global ranking has been based exclusively on three resilience traits (Varroa index, hygienic behavior, and Varroa reproduction). This adjustment was implemented because behavioral and productivity traits showed consistently high scores across years, whereas greater variation and potential for improvement were observed in resilience traits.

### 2.4. Statistics

Data analysis was conducted using RStudio version 4.4.2 (RStudio, Boston, MA, USA). Visualization was conducted in both RStudio and Microsoft Excel (Version 251 (Build 16.0.19426.20218)). To assess temporal trends in trait values, mixed models were fitted with the trait of interest as the response variable and testing year as a fixed effect. Beekeeper was included as a random intercept to account for the non-independence of colonies managed by the same beekeeper. Likelihood ratio tests were performed to evaluate the necessity of including the random effect. Model assumptions were assessed using simulation-based diagnostics, dispersion, AIC, etc. Depending on the nature and distribution of the response variable, different model types and transformations were applied. Behavioral traits were analyzed using mixed-effects models adapted to their scale. Swarming tendency, originally recorded as an ordinal variable (1–4) and strongly skewed towards the highest category, was recoded into a binary variable (score 4 vs. lower scores) and analyzed using a generalized linear mixed model (GLMM). In contrast, gentleness and calmness were analyzed using linear mixed-effects models (LMMs), as averaged scores resulted in approximately continuous response variables, after log-transformation to correct for right-skewness. The productivity traits were analyzed using LMMs. The spring development index showed a highly skewed distribution and was log-transformed before analysis. Resilience traits were analyzed using LMMs with trait-specific transformations where necessary. The winter index was modeled with year as a continuous predictor and a year-specific variance structure was included to account for heteroscedasticity. Varroa infection measures were log-transformed to correct for strong right-skewness. Hygienic behavior (24 h) data were left-skewed and therefore reflected and log-transformed using log(101 − x) to improve model fit. The subspecies was included as an additional fixed effect in supplementary mixed-effects analyses to assess its potential influence. The subspecies was not included in the primary models, because the dataset was highly unbalanced, with ≈90% of colonies belonging to the *Apis mellifera* *carnica* subspecies (see Descriptive Statistics).

To evaluate generational trends in performance (global ranking value and Q-value), multiple complementary approaches were used. The probability that an offspring has a lower global ranking value than its mother was analyzed using a GLMM (Binomial distribution; 1 = offspring lower than mother, 0 = not lower) with testing year as a categorical fixed effect and mother’s identity as a random intercept to account for repeated observations per mother. Differences in Q-values between mothers and their offspring were classified into three ordered categories (offspring lower, equal, or higher than the mother) and analyzed using a cumulative link mixed model (CLMM), including testing year as a categorical fixed effect and mother’s identity as a random intercept. For both models, the effect of testing year was assessed using likelihood ratio tests. To evaluate the impact of changes in the ranking methodology, an additional model including method version as a fixed effect was fitted and compared with the base model using a likelihood ratio test.

Trait-specific generational analyses were restricted to traits for which the testing protocol remained consistent throughout the study period, to ensure comparability across years. Associations between maternal and offspring performance for the Varroa index and spring development traits (ordinal categorical variables) were evaluated using Fisher’s exact test due to low expected cell counts. To assess directional changes, LMMs were fitted with the mother’s identity as a random effect. Although the response variable was ordinal, the scale was treated as approximately continuous due to evenly spaced categories. Maternal performance class (Q-value) was included as a fixed effect in the model to test whether offspring performance depended on maternal performance. Pairwise comparisons were conducted using estimated marginal means with Tukey-adjusted *p*-values, and contrasts against zero were used to test for significant deviations in performance between offspring and maternal within each class.

## 3. Results

### 3.1. Descriptive Statistics

Across the study period, 91 different beekeepers participated in the breeding program and a total of 2021 queens were tested, with a mean of ≈7 queens per beekeeper. The dataset contained 1825 *A. mellifera carnica* queens, 143 *Apis mellifera* Buckfast queens, 39 *Apis mellifera mellifera* queens and 14 queens of an unknown species. On average, 6 out of the 10 traits were assessed per queen. Of all tested queens, ≈70% were evaluated for all three behavioral traits, ≈35% for both productivity traits, ≈12% for all four resilience traits and ≈69% for the SOV trait. Trait availability varied across queens due to incomplete testing. The number of top queens varied between years, with an average of 48 queens per year and ranging from 38 to 63 queens. Among these, an average of 11 top queens per year had offspring that were tested in consecutive years. The global ranking values and Q-values of 229 mother-offspring pairs were compared. For the separate traits, Varroa index and spring development, Q-values of 215 mother-offspring pairs were compared. Detailed yearly numbers are provided in Supplementary Table S1.

### 3.2. Colony Performance Across Years

To assess improvement in colony performance over multiple years of mass selection, temporal trends were evaluated for each trait. First, the temporal trends of the behavioral traits, gentleness and calmness, were analyzed using log-transformed data and back-transformed for visualization (Supplementary Figures S2 and S3). Both traits showed significant positive temporal trends (gentleness: β = 0.018 ± 0.002 SE, *t* = 8.08, *p* < 0.001; calmness: β = 0.018 ± 0.002 SE, *t* = 8.52, *p* < 0.001), indicating increasing scores over time. Swarming tendency, analyzed as a binary variable (score 4 vs. lower scores), also increased significantly (β = 0.39 ± 0.11 SE, *z* = 3.72, *p* < 0.001), reflecting a higher probability of achieving the highest score over time (Supplementary Figure S4). Overall, all behavioral traits improved over time.

Second, the temporal trends of the productivity traits were assessed. For honey yield, no significant change (β = 0.13 ± 0.24 SE, *t* = 0.52, *p* = 0.60; Supplementary Figure S5) was observed. Similarly, no significant differences were detected in the honey index across years (2021–2024) (Supplementary Figure S6). In contrast, spring development increased significantly over time (β = 0.12 ± 0.01 SE, *t* = 8.72, *p* < 0.001; Supplementary Figure S7).

**Figure 1:**
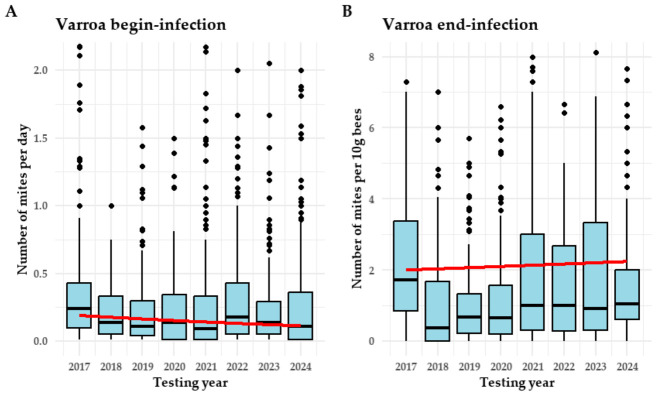
Boxplots of the Varroa begin-infection and Varroa end-infection results per testing year (2017–2024). (**A**) Varroa infestation at the beginning of the season is the average daily mite fall over approximately 21 days during early spring. (**B**) Varroa infestation at the end of the season, expressed as the number of phoretic mites collected using the sugar shake method on (standardized) 10 g of nurse bees in mid-summer. The red lines represent the overall trends estimated using mixed-effects models that account for repeated measurements within beekeepers.

**Figure 2:**
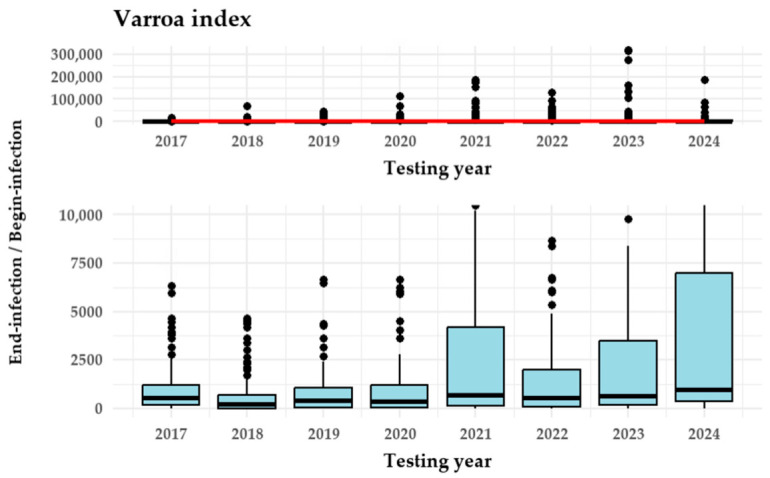
Boxplots of the Varroa index results (end-infection divided by begin-infection) per testing year (2017–2024). The upper panel shows the full range of the data, including the outliers, with the red line indicating the overall temporal trend estimated using a mixed-effects model that accounts for repeated measurements within beekeepers. Due to the wide range of values, variation within years is difficult to discern in this panel. The lower panel shows a zoomed-in view of the same data, excluding extreme values to better visualize the boxplot distribution. Note that the y-axis scale differs between panels.

**Figure 3:**
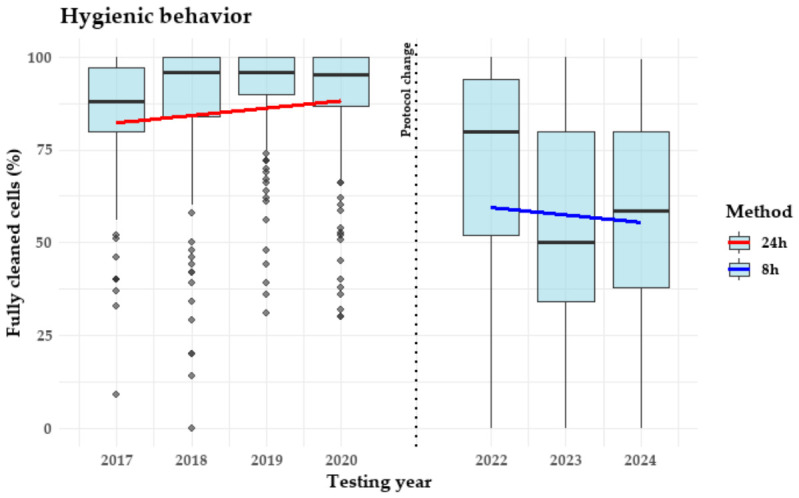
Boxplots of the hygienic behavior results per testing year (2017–2020 & 2022–2024), represented by the percentage of fully cleaned brood cells after being pin-killed. From 2017 to 2020, hygienic behavior was assessed after 24 h, whereas from 2022 to 2024, it was assessed after 8 h. This change in protocol is indicated by the vertical dotted line. The red line represents the temporal increasing trend for 2017–2020, and the blue line represents the decreasing trend for 2022–2024, both estimated using mixed-effects models that account for repeated measurements within beekeepers.

**Figure 4:**
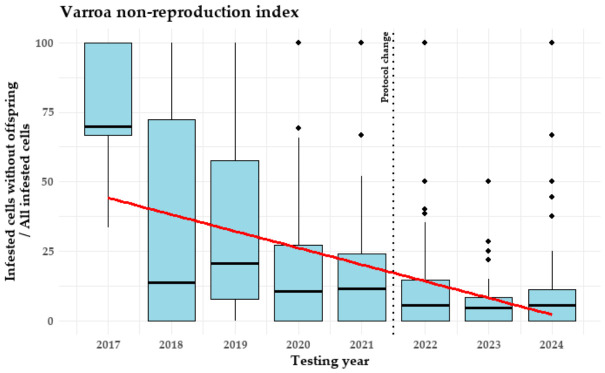
Boxplots of the Varroa non-reproduction index results per testing year (2017–2024), expressed as the proportion of infested cells without offspring relative to all infested cells. From 2017 to 2021, 200 cells per brood sample were examined for mite presence and reproduction, whereas from 2022 to 2024, inspections were stopped if no mites were detected within the first 30 cells. This change in protocol is indicated by the vertical dotted line. Despite this change in sampling effort, the calculation of non-reproduction remained consistent across years. The red line represents the overall decreasing trend estimated using a mixed-effects model that accounts for repeated measurements within beekeepers.

Lastly, the temporal trends of the resilience traits revealed varied outcomes. The overwintering index increased significantly over time (β = 0.91 ± 0.25 SE, *t* = 3.66, *p* < 0.001; Supplementary Figure S8). Variance differed between years, with higher variability observed in later years, particularly around 2020. Varroa begin-infection showed a significant decrease (β = −0.07 ± 0.02 SE, *t* = −3.70, *p* < 0.001; Figure 1A), while Varroa end-infection showed a non-significant positive trend (β = 0.016 ± 0.009 SE, *t* = 1.74, *p* = 0.08; Figure 1B). Overall, the Varroa index increased significantly over time (β = 0.29 ± 0.04 SE, *t* = 6.52, *p* < 0.001; Figure 2). Hygienic behavior scores were divided into two distinct models due to protocol changes during the study period. From 2017 to 2020, scores increased significantly (log(101 − x)-transformed data: β = −0.21 ± 0.04 SE, *t* = −4.72, *p* < 0.001), whereas no significant trend was observed from 2022 to 2024 (β = −1.99 ± 1.64 SE, *t* = −1.22, *p* = 0.22). Data from 2021 were excluded due to inconsistent methodology (Figure 3). The Varroa non-reproduction index decreased significantly over time (β = −6.00 ± 0.55 SE, *t* = −10.98, *p* < 0.001; Figure 4). For the derived parameters in 2022–2024, no significant trend was observed for MNR (β = −1.20 ± 0.83 SE, *t* = −1.44, *p* = 0.15), whereas mVR increased significantly (β = 0.61 ± 0.14 SE, *t* = 4.26, *p* < 0.001; Supplementary Figures S9 and S10). Including subspecies as an additional fixed effect did not alter the significance or direction of any temporal trends.

### 3.3. Colony Performance Across Generations

To assess generational changes in colony performance, ranking values and Q-values of top queens (mothers) were compared with those of their offspring across multiple years of mass selection. When pooling data across all years, offspring had a lower global ranking value than their mother in ≈79% of cases, while ≈4% retained the same value and ≈17% exceeded the mother’s ranking value. This pattern was consistent within individual testing years. After accounting for the non-independence of offspring from the same mother, the probability that offspring scored lower than their mother did not differ significantly between testing years (χ^2^(5) = 9.61, *p* = 0.09). Substantial variation was present between mothers in their tendency to produce lower-ranking offspring. The extent to which offspring and their mothers differed in ranking value varied across years. Compared to the reference year (2017), offspring from mothers tested in 2018 and 2019 had significantly lower global ranking values (*p* = 0.03 and *p* = 0.004, respectively), whereas differences in other years were smaller and not statistically significant. To evaluate the potential impact of changes in the ranking methodology (Table 2), the method was included as an additional factor. No significant effect was found (χ^2^ = 0.82, *p* = 0.67). However, the method and testing year were strongly confounded. A sensitivity analysis on a sub-dataset (2017–2020), where method variation was more balanced, showed a significant effect of testing year (χ^2^(3) = 9.38, *p* = 0.03), suggesting that temporal effects were not solely driven by methodological changes.

Similar patterns were observed when comparing Q-values between mothers and their offspring. When pooling data across all years, offspring had lower Q-values than their mother in ≈63% of cases, while ≈31% were equal and ≈5% were higher. Mixed model analysis showed no significant effect of testing year on the distribution of these outcomes (χ^2^(5) = 2.75, *p* = 0.74), suggesting the testing year had no detectable influence on the difference between the Q-values of the offspring and their mothers. Inclusion of the (ranking) method did not improve model fit (χ^2^(2) = 0.08, *p* = 0.96), and similar confounding between method and testing year was observed. These findings were consistent with the sensitivity analysis described above.

**Figure 5:**
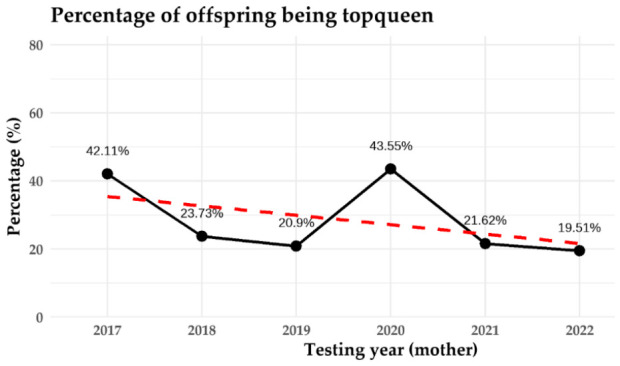
Percentage of offspring from top queens that are also classified as top queens. The *x*-axis indicates the year in which the mother queen was tested, and the y-axis shows the proportion of her offspring achieving top queen status.

Ultimately, knowing the offspring’s ranking and Q-values allows us to evaluate the proportion of offspring (bred from top queens) who are also top queens. When pooling data across all years, ≈29% of offspring were classified as top queens. Figure 5 shows that the proportion of top-performing offspring remained below 50% in all years, indicating that fewer than half of the offspring of top queens are top queens themselves.

Next, we investigated whether offspring performance within individual traits differed from that of their mothers (top queens) by comparing Q-values. Even though the mother was a top queen (with a Q-value of 1.00 or 0.75 based on global ranking score), for a specific trait, she could have had any Q-value (1.00–0.25). Analyses were restricted to traits with consistent protocols over time.

**Figure 6:**
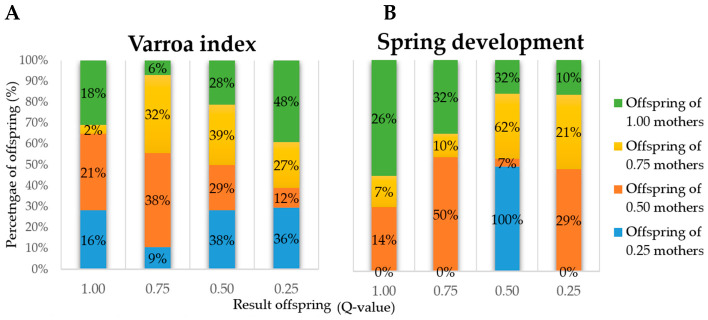
Distribution of offspring per Q-value, of different maternal Q-value categories for (**A**) the Varroa index and (**B**) spring development. Bars represent the percentage of offspring assigned to each Q-value (1.00–0.25), with colors indicating the Q-value of the mother (top queen). The legends show the different maternal categories. Q-values correspond to quarter-based rankings, where 1.00 represents the highest-performing queens and 0.25 the lowest.

Figure 6A shows the distribution of offspring per Q-value, descending from top queens with different Q-values for the Varroa index trait. Generally, most offspring of lower-scoring mothers (Q-value = 0.50 and 0.25) had a better result than their mother, while offspring of higher-scoring mothers (Q-value = 1.00 and 0.75) had a worse result. For example, the highest proportion of offspring (38%) from 0.50 mothers had a result in the second quarter (Q-value = 0.75). A significant association was observed between maternal and offspring Q-values (*p* < 0.001), indicating that offspring performance was not independent of the maternal performance. However, there was no significant overall difference between offspring and maternal Q-values (β = −0.08 ± 0.06 SE, *t* = −1.37, *p* = 0.18), indicating that offspring did not consistently score higher or lower than their mothers. The extent of the difference between offspring and maternal Q-values varied significantly across maternal Q-value categories (*p* < 0.001). Post hoc comparisons (Tukey-adjusted) showed that offspring from queens with a Q-value of 1.00 exhibited a larger decline compared to those from 0.25 (estimate = 0.87 ± 0.07 SE, *t* = 12.96, *p* < 0.001), 0.50 (estimate = 0.70 ± 0.09 SE, *t* = 7.81, *p* < 0.001), and 0.75 queens (estimate = 0.36 ± 0.09 SE, *t* = 4.18, *p* < 0.001). Similarly, offspring from 0.75 queens showed a greater decline than those from 0.25 (estimate = 0.52 ± 0.09 SE, *t* = 5.84, *p* < 0.001) and 0.50 queens (estimate = 0.34 ± 0.10 SE, *t* = 3.36, *p* = 0.01), while the difference between 0.25 and 0.50 queens was not significant (estimate = 0.17 ± 0.09 SE, *t* = 1.86, *p* = 0.263). When comparing offspring directly to their mother within each maternal category, offspring of lower-scoring queens had significantly higher Q-values than their mothers (0.25: estimate = 0.35 ± 0.05 SE, *t* = 6.48, *p* < 0.001; 0.50: estimate = 0.18 ± 0.08 SE, *t* = 2.40, *p* = 0.02), whereas offspring of higher-scoring queens had significantly lower Q-values (0.75: estimate = −0.16 ± 0.07 SE, *t* = −2.35, *p* = 0.02; 1.00: estimate = −0.52 ± 0.05 SE, *t* = −10.59, *p* < 0.001). Overall, these results indicate a clear shift in offspring performance across mother categories: offspring of lower-scoring queens tend to outperform their mothers, whereas offspring of higher-scoring queens tend to underperform relative to their mothers, with the magnitude of this difference varying between categories.

Similar patterns are seen for spring development (Figure 6B), with, for example, 50% of the offspring from 0.50 mothers that had a result in the second quarter (Q-value = 0.75). A significant association between maternal and offspring Q-values was detected (*p* = 0.002). In contrast to the Varroa index, offspring showed a significantly lower Q-value than their mothers on average across all mother-offspring comparisons (β = −0.20 ± 0.06 SE, *t* = −3.29, *p* = 0.004). The extent of the difference between offspring and maternal Q-values varied significantly across maternal categories (*p* = 0.01). Post hoc comparisons indicated that offspring from 1.00 mothers exhibited a significantly larger decline compared to those from 0.50 queens (estimate = 0.50 ± 0.15 SE, *t* = 3.51, *p* = 0.01), while no other pairwise differences were significant. Within-category comparisons showed no significant differences for offspring of lower-scoring queens (0.25: estimate = 0.25 ± 0.26 SE, *t* = 0.97, *p* = 0.34; 0.50: estimate = 0.16 ± 0.13 SE, *t* = 1.28, *p* = 0.22). In contrast, offspring of higher-scoring queens had significantly lower Q-values (0.75: estimate = −0.21 ± 0.08 SE, *t* = −2.50, *p* = 0.02; 1.00: estimate = −0.34 ± 0.07 SE, *t* = −4.93, *p* < 0.001). Overall, these results indicate that offspring of higher-scoring queens tend to underperform relative to their mothers, whereas offspring of lower-scoring queens do not differ significantly from their mothers.

## 4. Discussion

At HBV, the selective breeding program aimed to improve overall population performance, ensuring that each successive generation showed improvements over the previous one, ideally across all traits of interest, but most importantly for the Varroa resilience traits.

### 4.1. Colony Performance over the Years

Upon initial analysis of the behavioral trait results—gentleness, calmness, and swarming tendency—the statistical models indicated an improvement over the years. Gentleness and calmness are highly genetically correlated [24,25,26]. Their strongly correlated response suggests that selection for increased gentleness is likely to result in a concurrent increase in calmness [27]. Furthermore, swarming tendency is also genetically well correlated with both gentleness and calmness [24,25]. Despite the observed increases in behavioral scores, inspection of the figures (Supplementary Figures S2–S4) suggests that this improvement has reached a plateau, with most colonies scoring between 3 and 4 and very few receiving scores of 1 or 2. This pattern is likely attributable to pre-selection by beekeepers during the testing season. These traits become evident early in the testing season, enabling beekeepers to make informed decisions when selecting colonies for further trait testing. For example, a colony exhibiting aggressive behavior, a tendency to swarm, and no steadiness on the comb will be excluded from the selection process by the beekeeper, and no additional traits will be evaluated in this study. Hence, data from these colonies were not submitted to the online administrative data tool, Breed It.

For productivity traits, statistical models indicated an upward trend over time for both honey yield and honey index; however, these trends were not statistically significant and appeared minimal in the corresponding figures (Supplementary Figures S5 and S6). Rather, honey yield results fluctuated over time, but no discernible improvement was evident. Although spring development increased significantly, similar to honey yield, this trend was not readily apparent. These traits are influenced by environmental factors, such as favorable weather and the proximity of flowering plants, as well as beekeeping management practices, including the relocation of hives for pollination and providing bees with adequate space within their hives [28,29]. For instance, colonies situated in a barren landscape with inadequate floral resources will produce less honey than colonies placed in an area with an abundance of melliferous plants, irrespective of their genetic background [27,30]. Thus, it is important to filter out environmental effects [27]. Since 2021, honey yield has been standardized at the apiary level and expressed as the relative contribution of a colony to the apiary average (honey index). Similar standardization was later applied to spring development, but these results are not included here, as the application was implemented only in the last two years of the study period.

In addition to behavior and productivity, the main objective of our breeding program was to enhance the resilience of our colonies, as demonstrated by the global ranking criteria introduced in 2021 and later in 2023. The winter index results showed a modest increase over time; however, its reliability has diminished due to the difficulty in timing the assessment. Climate change has resulted in warmer autumns, causing a delayed reaction in winter bees to form a winter cluster [31]. The Varroa index results increased over the years, which is unfavorable, as it indicates an increasing mite population growth during the testing season. This index is derived from two separate measurements (Varroa begin- and end-infection). The begin-infection decreased over time, suggesting that fewer mites were present at the beginning of the season. However, this low number may reflect the effectiveness of winter chemical treatments applied by beekeepers [32]. In contrast, the end-infection in summer is more informative for estimating phoretic mite levels within the hive, as it is expected to be less influenced by beekeepers’ interventions against *Varroa* (if participating beekeepers followed our recommendations of not chemically treating during the testing season). However, this estimation does not take into account the mite levels in the brood. Notably, the results indicated that phoretic mite infection levels in summer increased over time. This suggests a worsening of within-season mite dynamics. However, some technical factors can restrict the measurement of mite infestation, such as the removal of mites by the powder sugar method due to small deviations in humidity or the environment that affects mite population growth and brood development [25]. Hygienic behavior, assessed by pin-killing brood, reflects both general and Varroa-specific (i.e., specifically Varroa-infested cells are cleaned) hygienic responses, as removal rates of pin-killed brood and mite-infested brood are positively correlated [33]. Hygienic scores increased during the early years of the breeding program, with the most pronounced improvement observed between 2017 and 2018 (Figure 3). From 2018 to 2020, scores stabilized, suggesting a plateau was reached. In response, the protocol was adjusted in 2022 to an 8 h assessment period, as different results were only then observed. Similar findings were reported by Hoppe et al. [25], where the waiting times for the pin test have been significantly reduced from the previous 24 h. Despite this adjustment, hygienic behavior scores decreased (non-significantly) in recent years. Finally, the Varroa non-reproduction index (~SMR) decreased significantly over time. In addition, the derived parameters showed similar negative results; the decreasing MNR scores (non-significant) indicated more reproducing mites per SIC, and the increasing mVR results denoted higher offspring production per foundress mite, per infested cell.

By selecting for multiple traits simultaneously, the breeding program aimed to prevent the improvement of one trait at the expense of others [25]. However, despite assigning greater weight to resilience-related traits, their improvement remained limited compared to the other traits. Breeding programs focusing on similar traits have comparable findings, whereby the relative increase in trait scores was honey yield, gentleness, calmness, swarming, hygienic behavior and Varroa infestation development [25]. Differences in the selection response may be explained by trait heritability. Traits with higher heritability—i.e., a stronger genetic component relative to environmental influence—typically achieve faster progress much more easily [34]. In contrast, traits with lower heritability are more strongly influenced by the environment, which can obscure genetic progress. Although the participating colonies were all located within Flanders and thus assumed to experience broadly similar environmental conditions, local variation in forage availability, weather, and management practices may still have contributed to phenotypic variation. In this study, heritability estimates were not computed, as this was beyond the primary scope of this research. Therefore, we relied on values reported in the literature, particularly for *Apis mellifera carnica*, the predominant subspecies in this breeding program. Previous studies have indicated that behavioral traits, especially gentleness and calmness, generally exhibit higher heritability than productivity traits, although all show sufficient genetic variation to allow selection progress [24,35]. Traits associated with Varroa resistance are crucial selection criteria; however, they frequently exhibit variability in their genetic characteristics. Hygienic behavior, as assessed by the pin test, typically shows a higher heritability than mite population development (VID ~Varroa index), but both traits show good prospects for response to selection [25,26,33,36]. Traits with lower heritability, such as Varroa index, generally require more generations to produce significant genetic growth [25,34]. In contrast, SMR exhibits relatively high heritability, allowing for advancement by targeted selection [37]. However, this is not reflected in our results. Over the years, we experienced difficulties with this trait due to the low quality of brood samples and the low number of (single) infested cells in these samples, causing inaccurate calculations [20]. Several of these traits are genetically correlated, supporting the use of a multi-trait selection approach. For example, hygienic behavior has been reported to correlate positively with both honey yield [26] and SMR [37]. Importantly, resistance to *V. destructor* is not determined by a single trait but rather by a combination of mechanisms that interact within a given environmental context [37].

### 4.2. Colony Performance over Generations

The dataset of generations is limited because of the implemented selection schedule, whereby a queen is tested in the first year, her daughter queens are produced in the second year, and these daughters are tested in the third year. Consequently, comparisons between generations cannot be made in consecutive years but must have a one-year interval. Furthermore, the analyses over generations were restricted to offspring of top queens, as these queens constituted the breeding stock within the program. Therefore, the results primarily reflect the effectiveness of the selection strategy among selected breeding lines rather than the breeding population as a whole. Although this approach may reduce the representativeness of the dataset, it provides the most direct assessment of whether the queens targeted for breeding produced offspring with improved performance.

Overall, the performance of offspring, in terms of the global ranking value, tended to be lower than that of their mother (≈79% of cases), while only a small proportion of offspring achieved similar (≈4%) or higher (≈17%) ranking values, and this trend was consistent for every individual testing year. Neither the testing year nor the ranking methodology had a significant effect on the comparison between mothers and offspring, indicating that global ranking values were comparable across years despite methodological adjustments. When considering the lack of independence among offspring from the same mother, the probability that offspring scored lower than their mother remained consistent across testing years. This indicates that in any given year, it was not the case that a significant number of offspring scored lower than their mothers. In addition, substantial variation was observed between mothers in their tendency to produce lower-ranking offspring, suggesting that, apart from mother-offspring genetic transmission of desirable traits, other factors, such as mating or environment, can influence how traits are passed on. The magnitude of the difference in ranking values between mothers and offspring also varied across years, with more pronounced declines observed for offspring of mothers tested in 2018 and 2019. This pattern could reflect temporal differences in selection intensity or in the genetic distinction between selected queens and the broader breeding population.

The distribution of global ranking results changed each year, meaning that a lower-ranking score for the offspring could still indicate that they were ranked as a top queen. This depends on the global ranking scores of all queens tested in that year. We therefore compared the Q-values of the mother with those of her offspring. These analyses showed similar patterns: across all years, the majority of offspring exhibited lower Q-values than their mothers (≈63%). In the next most prevalent scenario (≈31%), the offspring exhibited identical Q-values to those of their mothers. Conversely, the least frequent occurrence involved offspring possessing higher Q-values than their mothers (≈5%). Similarly, testing year and ranking methodology had no significant effect on the comparison of Q-values between mother and offspring. To determine whether a tested queen was a top queen, we examined in which quarter the global ranking score fell (See Table 1). If it fell in the first or second quarter, with Q-values of 1.00 and 0.75, respectively, we concluded that the queen was a top queen. Throughout all testing years, fewer than half of the offspring of top queens also became top queens (Figure 5).

The limited performance improvement observed in the individual traits over time is reflected in the overall performance of queens across generations. These unexpected results may be explained by various factors regarding the establishment and operation of the selection program. First, every year, participation in the program was open to all beekeepers, regardless of prior experience in selection or the genetic quality of their starting material. While this approach promotes accessibility, it may introduce selection bias and substantial variability in baseline colony performance. Some beekeepers purchase daughter queens of stocks proven to have desirable traits as a starting point, whereas others use their existing (non-selected) stock, potentially diluting overall selection intensity. Although this continuous influx of new genetic material increases genetic diversity and can thereby reduce inbreeding, it can simultaneously slow selection progress if lower-quality colonies are incorporated into the breeding population [16]. A potential approach to mitigate this issue would be to subdivide the breeding population into two groups: an established population, including queens from lines that have undergone selection over multiple generations, and a secondary group consisting of newly introduced, unselected colonies. Integration of the latter into the established population should be contingent on several years of demonstrated progress in selection, thereby safeguarding the overall genetic quality of the breeding population.

Second, the effectiveness of selection may have been limited by the absence of strict, standardized criteria for the setup and evaluation of participating colonies. Such criteria may include exchanging colonies among beekeepers to reduce evaluator bias, testing a minimum number of colonies per year, and including genetically related colonies (e.g., sister queens) to allow more reliable comparisons. In addition, maintaining all test colonies of a given beekeeper within the same apiary can help minimize environmental variation. Finally, knowledge of the maternal background—preferably including prior performance testing—would improve the reliability of selection decisions. Furthermore, verifying adherence to breeding recommendations, focusing on targeted traits, could improve selection consistency. The absence of such standardized procedures in the present program may have increased data variability and reduced overall selection efficiency.

In addition to program-related factors, genetic mechanisms may also contribute to the observation that offspring of top-performing queens often failed to achieve performance levels comparable to those of their mothers. Many honey bee traits, such as hygienic behavior and MNR, are complex quantitative traits that are influenced by multiple genes (polygenic) and their combined effects [38]. Furthermore, the expression of some favorable genetic variants may depend on recessive alleles; however, homozygosity for recessive alleles is difficult to achieve due to the polyandrous mating system of the honey bee queen. Moreover, the honey bee genome is characterized by a high recombination rate [39]. As a result, substantial variation may arise both between mothers and their offspring and among offspring descending from the same queen. In addition, part of the phenotypic variance can be attributed to non-additive genetic effects such as dominant effects and/or epistasis [38]. Collectively, these factors complicate selection for these traits. Genomic mapping and application of marker-assisted selection may help overcome some of these challenges [40].

Closely related to these genetic mechanisms, paternal genetics plays a crucial role in shaping honey bee populations and likely constitutes a constraint in this selection program. Although breeding decisions were made intentionally, with beekeepers receiving recommendations based on the presence of desirable traits in the selected colonies, mating itself was not strictly controlled. While important for attaining sustainable breeding progress, mating control requires more effort [41,42]. Rather than providing direct mating instructions, advice focused on which traits to strengthen in particular queen lines. Consequently, beekeepers who chose to use mating stations (land or island) were encouraged to select stations containing drone lines with complementary characteristics to enhance the genetic potential of virgin daughter queens further [43]. In this way, controlled mating allows breeders to influence both the maternal and paternal genetic contribution to the colony [34]. However, participation in controlled mating remained optional, and free mating was also permitted within the program. As a result, queens could mate with multiple drones from surrounding, largely unselected populations, potentially counteracting the genetic gains achieved through maternal selection [44]. This effect is particularly pronounced in densely populated beekeeping regions such as Flanders, where controlling the mating environment is inherently difficult. Relying exclusively on controlled mating stations is unlikely to provide a complete solution, as excessive restriction of mating populations may reduce genetic diversity [25]. An alternative within a closed breeding system is instrumental insemination (II), which allows precise control over the combination of selected queens and drones [30,41]. However, due to the requirement for specialized equipment and technical expertise, this approach is currently feasible only on a limited scale and primarily for more experienced breeders [30]. Such controlled mating strategies may therefore be particularly suitable for beekeepers working with selection lines that have undergone multiple generations of selection, referring to the established population group mentioned earlier. Still, completely abandoning open breeding systems, in which queens mate naturally, is not a viable approach, because this type of mating is more accessible to a broader range of beekeepers. Furthermore, traits can improve within selection programs that allow natural mating [41,45,46]. However, under such conditions, additional measures are required to prevent the loss of genetic progress from (only) selective breeding. One possible strategy would be to introduce selected drone-producing colonies throughout Flanders, preferably originating from the previously described established breeding population. By increasing the frequency of desirable drones in the mating environment, this approach could enhance favorable traits in naturally mated queens [30]. Thus, a combined mating structure could be considered, whereby experienced breeders with established selection lines apply controlled mating practices, whereas less experienced breeders with newly introduced populations continue with free mating with selected drone-producing colonies in their region.

Finally, selection decisions were based on phenotypic performance rather than estimated breeding values. Consequently, queens could be classified as top queens in a given year without accounting for the genetic background, i.e., the phenotypic performances of previous generations. Incorporating breeding values allows for a more reliable identification of genetically superior individuals and could enhance long-term selection response.

Interestingly, when the Q-values of individual traits (Varroa index and spring development) were evaluated, offspring performance appeared to depend strongly on the maternal Q-value category (Figure 6). Offspring from lower-scoring queens (Q-values = 0.25 or 0.50) generally performed better than their mothers, whereas offspring from higher-scoring queens (Q-values = 0.75 or 1.00) frequently showed reduced performance. This pattern was particularly evident for the Varroa index and, to a lesser extent, spring development. A previous study using data from the HBV selection program [19] showed that, for specific traits, queens from the fourth quarter (Q-value = 0.25) exhibited a substantially higher mortality rate than queens from the other quarters. Moreover, for hygienic behavior (24 h) and Varroa index, mortality rates were comparable among queens from the first, second, and third quarters, while for spring development, similar mortality rates were observed for queens from the first and second quarters. These findings suggest that colonies with the highest scores for certain traits do not necessarily have a greater likelihood of winter survival [19]. In combination with the present results, showing that offspring from top-scoring queens (for the overall ranking, but also for some individual traits) do not necessarily achieve scores comparable to their mothers (indeed, they often score lower), it may be more advantageous to implement a breeding strategy that excludes the lowest-scoring colonies rather than continuing to breed from the highest-scoring ones. This strategy, referred to as selection by exclusion (i.e., negative selection), is closely related to the natural way of selection, in which the fittest colonies develop well, while the weakest stagnate and are removed [10]. This approach may also help maintain a broader genetic pool, thereby supporting the capacity of honey bees to mitigate the effects of parasitism [44]. Future breeding programs in Flanders should evaluate whether selection by exclusion represents a practical and effective alternative to conventional mass selection and whether it can improve long-term honey bee colony performance and adaptability to current and future stressors.

## 5. Conclusions

The Honeybee Valley selection program was established as an accessible local breeding initiative that beekeepers with diverse backgrounds could participate in. Selection focused on multiple traits, with particular emphasis on improving the resilience of Flemish honey bee populations. Over time, greater focus was placed on these Varroa-related resistance traits, as progress in these traits appeared limited. The present evaluation showed that progress was achieved mainly for behavior and productivity traits, whereas improvements in resistance-related traits remained modest. Furthermore, offspring of selected top-ranking queens frequently failed to achieve performance levels comparable to those of their mothers; in most cases, they performed worse, indicating limited cumulative genetic progress across generations. These findings suggest that the current implementation of mass selection may contain inherent limitations that reduce overall selection efficiency. Future breeding efforts in Flanders may therefore benefit from stricter selection criteria, improved mating control, and greater adherence to breeding recommendations, while maintaining sufficient genetic diversity and accessibility for beekeepers. Alternative strategies, such as selection by exclusion, may also represent a promising approach for achieving more sustainable improvements in honey bee health and resilience.

## Figures and Tables

**Table 1:** Distribution of different quarters with the corresponding Q-values and ranking results.

Quarter	Q-Value	Result
First	1.00	Best
Second	0.75	
Third	0.50	
Fourth	0.25	Worst

**Table 2:** Overview of different global ranking methods in the mass breeding program (2017–2024).

Method	Testing Years	Formula	Criteria
A	2017–2020	$=\left(0.25\times Q_{behavior}+0.25\times Q_{productivity}+0.50\times Q_{resilience}\right)\times 100$	SOV+
B	2021–2022	$=\left(0.25\times Q_{behavior}+0.25\times Q_{productivity}+0.50\times Q_{resilience}\right)\times 100$	SOV+ Min. of three resilience traits tested
C	2023–2024	$=\left(\frac{Q_{VI}+Q_{HY}+Q_{VR}}{\#\;performed\;tests}\;\right)\times 100$	SOV+ VI, HY and VR performed

Q = Q-value corresponding to the quarter in which the test result falls; VI = Varroa index; HY = hygienic behavior; VR = Varroa reproduction; SOV+ = SOV-positive (no viruses present).

## Data Availability

The data presented in this study are available upon request from the corresponding author. The data are not publicly available due to privacy restrictions.

## Supplementary Materials

The following supporting information can be downloaded at https://www.mdpi.com/article/10.3390/insects17070689/s1. Figure S1: Schematic overview of the workflow of the selective breeding program at Honeybee Valley; Figure S2: Boxplots of the gentleness scores per testing year (2017–2024); Figure S3: Boxplots of the calmness scores per testing year (2017–2024); Figure S4: Predicted probabilities of swarming tendency highest score (4) per testing year (2017–2024); Figure S5: Boxplots of the honey yield per testing years (2017–2024); Figure S6: Boxplots of the honey index (per apiary) per testing year (2021–2024); Figure S7: Boxplots of the spring development scores per testing year (2017–2024); Figure S8: Boxplots of the winter index per testing year (2017–2024); Figure S9: Boxplots of the mite non-reproduction scores per testing year (2022–2024); Figure S10: Boxplots of the mean Varroa reproduction rate scores per testing year (2022–2024); Table S1: Representativeness of the data collection over testing years.

## Abbreviations

The following abbreviations are used in this manuscript:

SOV	Suppressed in ovo virus infection
DWV	Deformed wing virus
BQCV	Black queen cell virus
ABPV	Acute bee paralysis virus
SBV	Sacbrood Virus
VSH	Varroa-sensitive hygiene
SMR	Suppressed mite reproduction
MNR	Mite non-reproduction
mVR	Mean Varroa reproduction
MICs	Multiple infested cells
SICs	Single infested cells
VI	Varroa index
HY	Hygienic behavior
VR	Varroa reproduction
GLMM	Generalized linear mixed model
LMM	Linear mixed-effects model
CLMM	Cumulative link mixed model
AIC	Akaike Information Criterion
SE	Standard Error
VID	Varroa infestation development
II	Instrumental insemination

## Appendix A

### Phenotypical Analysis of Brood Samples

Brood cells were opened and data were collected on the number of mother mites, as well as the number of male and female offspring present in each cell. Next, different phenotypes were classified: no mother mite present (Phenotype A), one mother mite present with no offspring (Phenotype B), one mother mite present with offspring (SICs) (Phenotype C) and multiple mother mites present with offspring (MICs) (Phenotype D). The protocol for the phenotypical analysis was grouped into different counting phases, whereby counting was stopped if too few mites were found in the opened brood cells. In this way, it is ensured that less time is spent on each brood sample if the infection rate is insufficient. If enough mites were found per phase, the counting continued to the next phase until a maximum of 350 cells were opened. If before the last phase, 35 SICs were found, counting was also halted.
