# Supplementary material for: Reconsidering the Selection Strategy in a Flemish Honey Bee Breeding Program: Towards Selection by Exclusion
Source: Insects. 2026 Jul 2;17(7):689. doi: 10.3390/insects17070689 (PMC13411069; doi:10.3390/insects17070689)
Supplement: Supplementary file 1 [file insects-17-00689-s001.zip › insects-4374830-supplementary.pdf]

Supplementary Information

Reconsidering the Selection Strategy in a Flemish Honey Bee Breeding Program: Towards Selection by Exclusion

Emma Bossuyt [1,*], Ellen Danneels [1], Dirk C. de Graaf [1,2]

Supplementary Figure S1. Schematic overview of the workflow of the selective breeding program at Honeybee Valley.

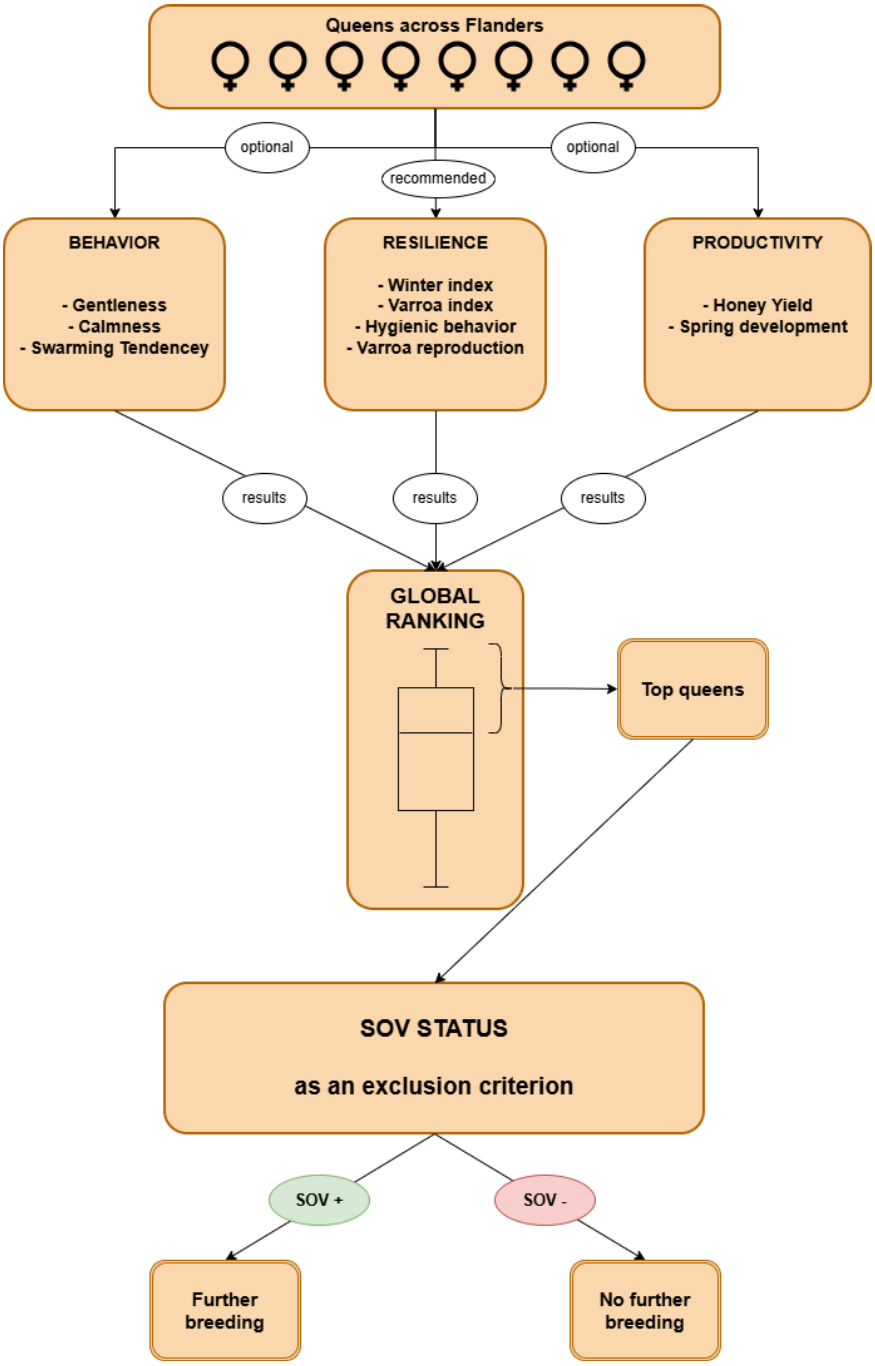

Supplementary Figure S2. Boxplots of the gentleness scores per testing year (2017-2024).

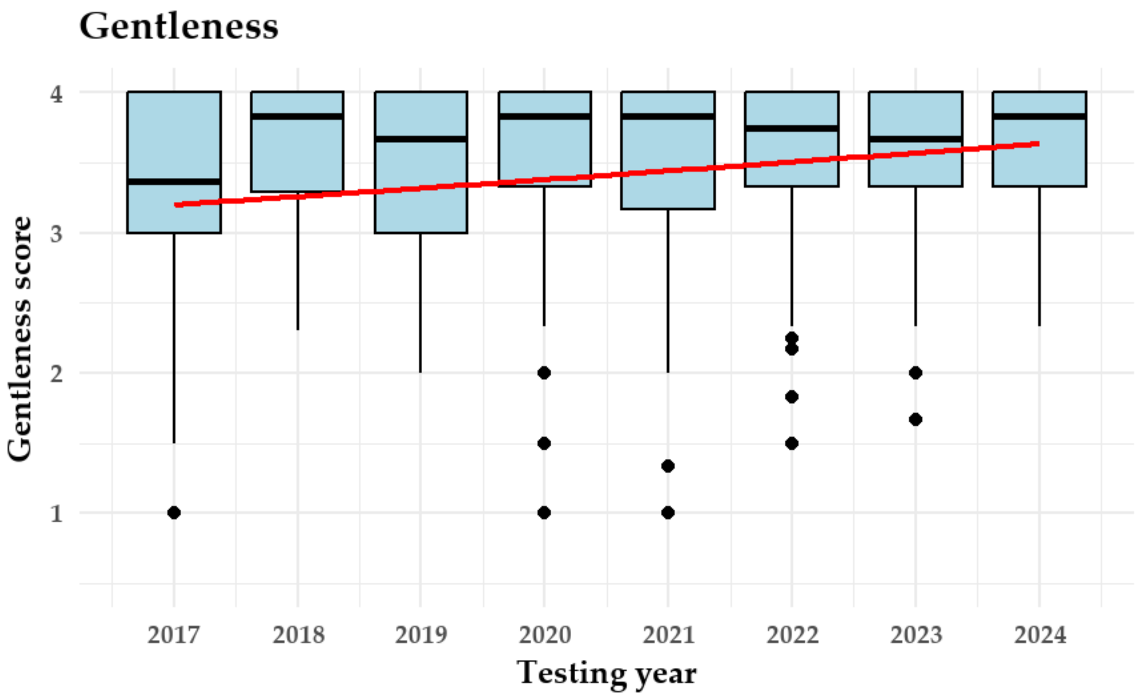

Supplementary Figure S3. Boxplots of the calmness scores per testing year (2017-2024).

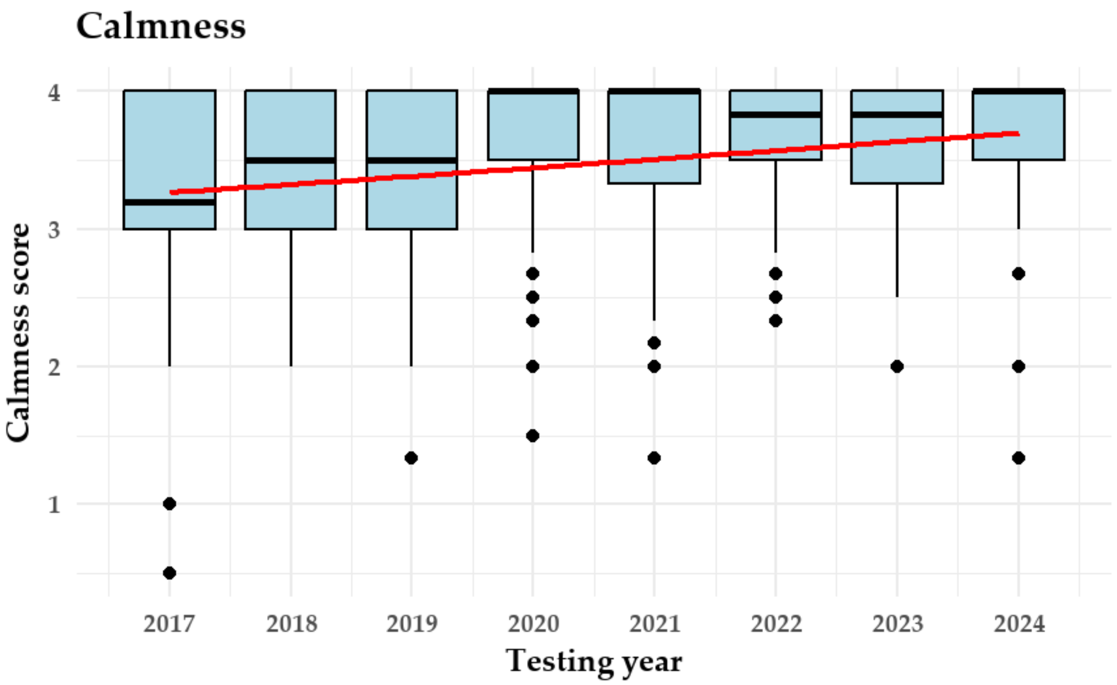

Supplementary Figure S4. Predicted probabilities of swarming tendency highest score (4) per testing year (2017-2024). Black dots are the observed proportion of score 4. Red line is the model prediction: upward trend over time

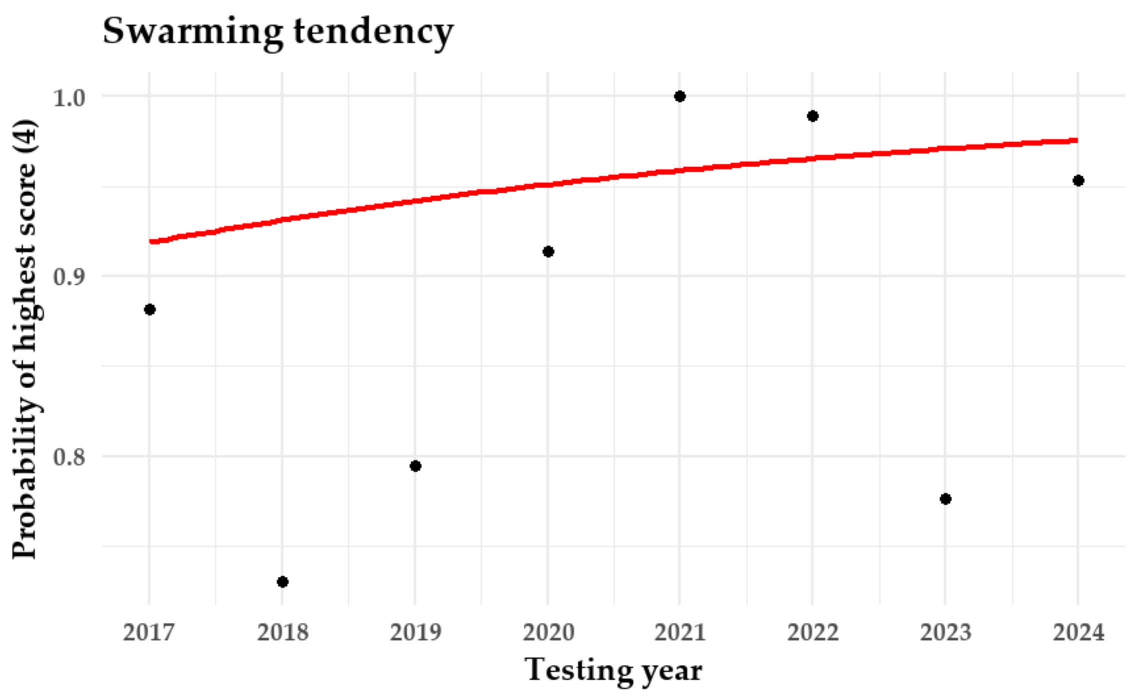

Supplementary Figure S5. Boxplots of the honey yield per testing year (2017-2024).

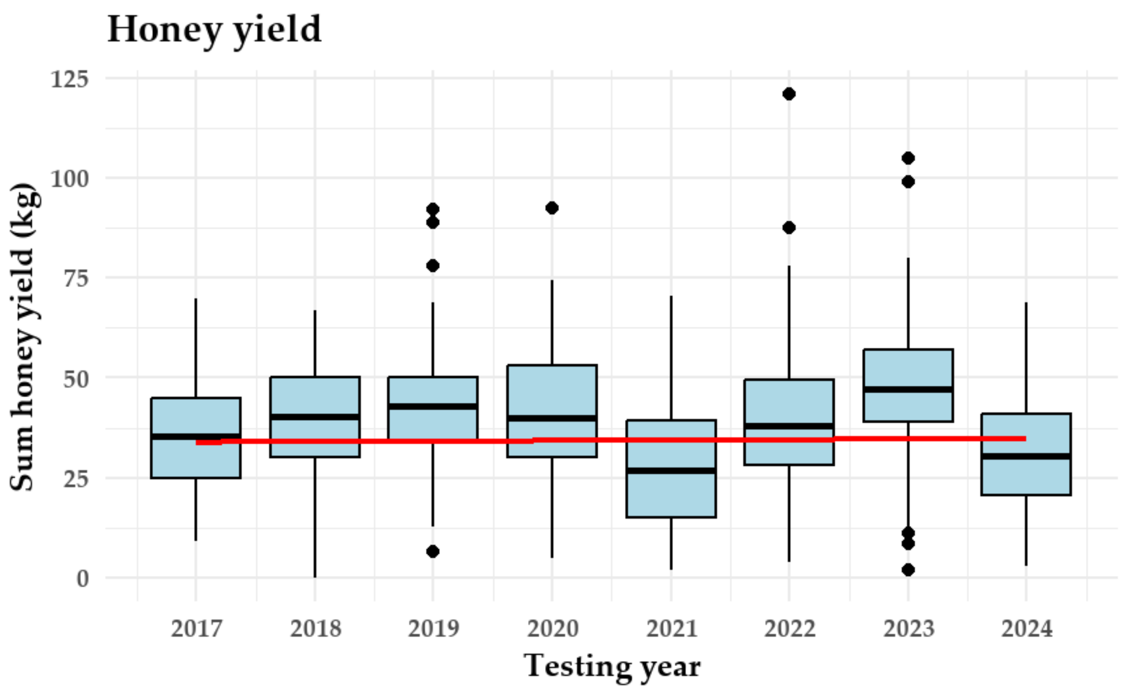

Supplementary Figure S6. Boxplots of the honey index (per apiary) per testing year (2021-2024).

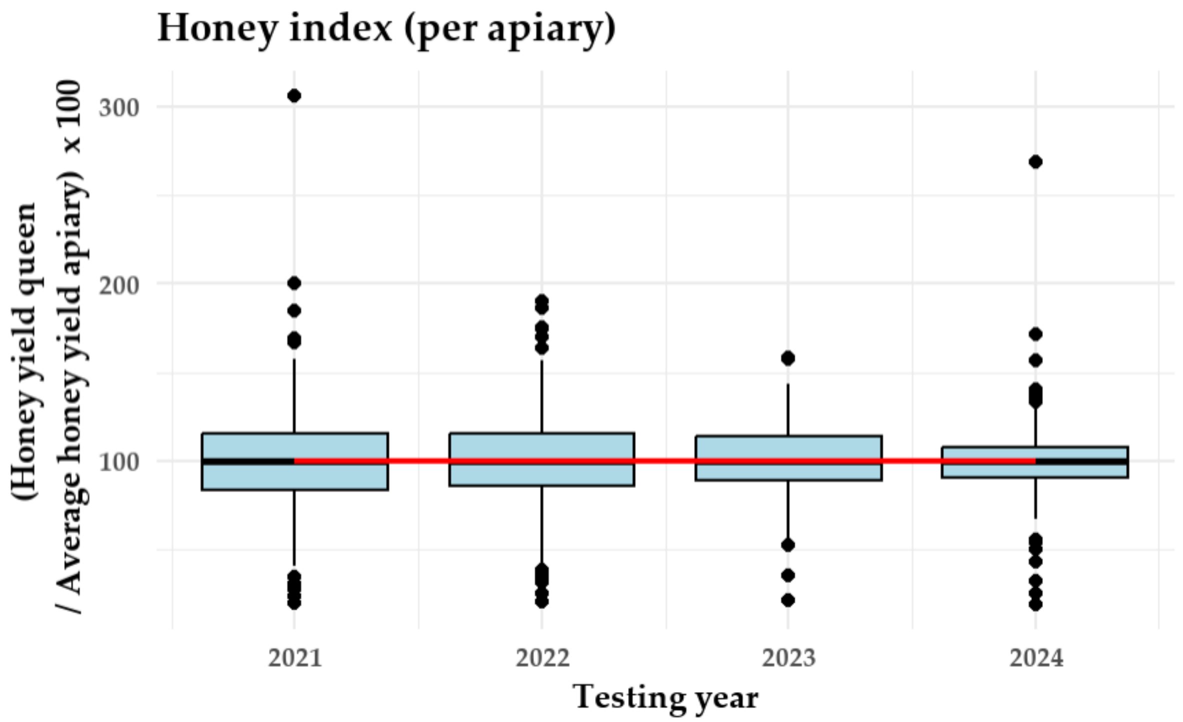

Supplementary Figure S7. Boxplots of the spring development scores per testing year (2017-2024).

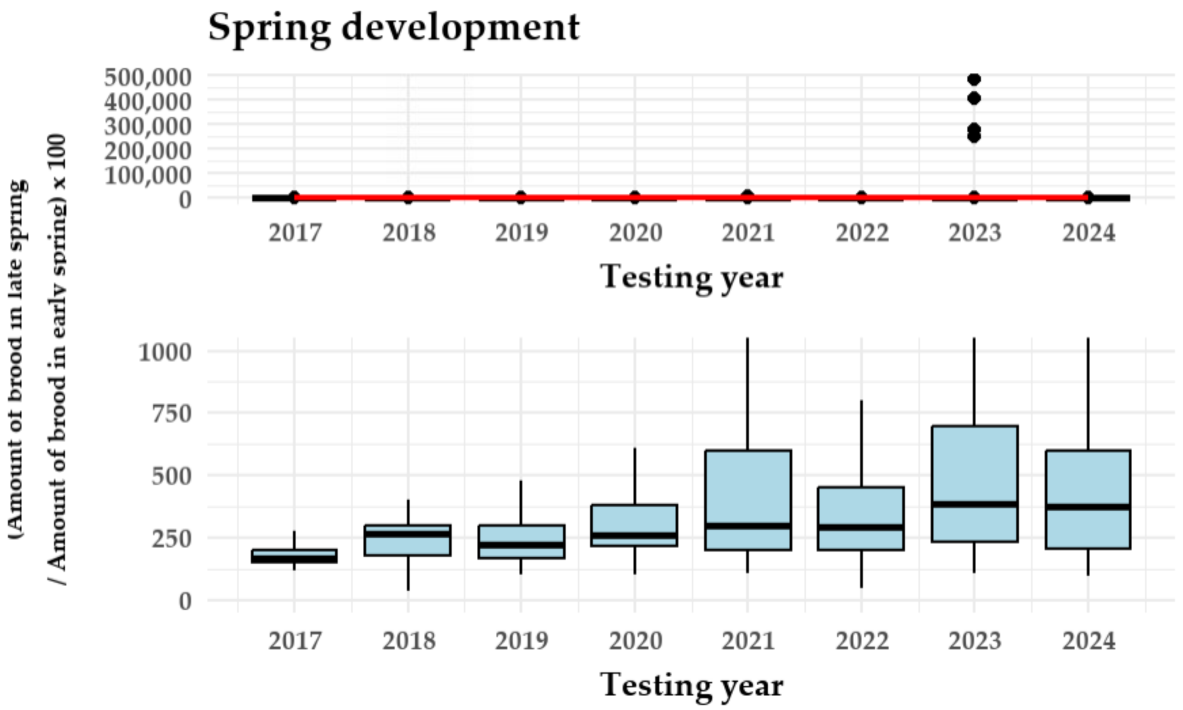

Supplementary Figure S8. Boxplots of the winter index per testing year (2017-2024).

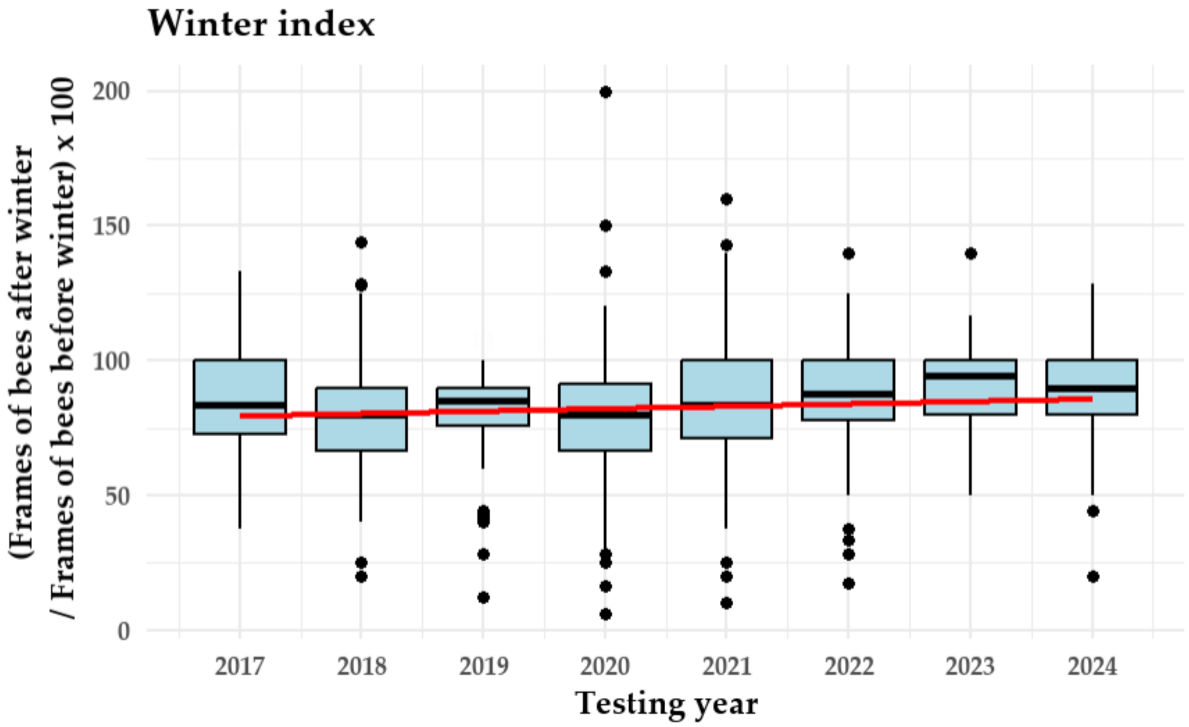

Supplementary Figure S9. Boxplots of the mite non-reproduction scores per testing year (2022-2024).

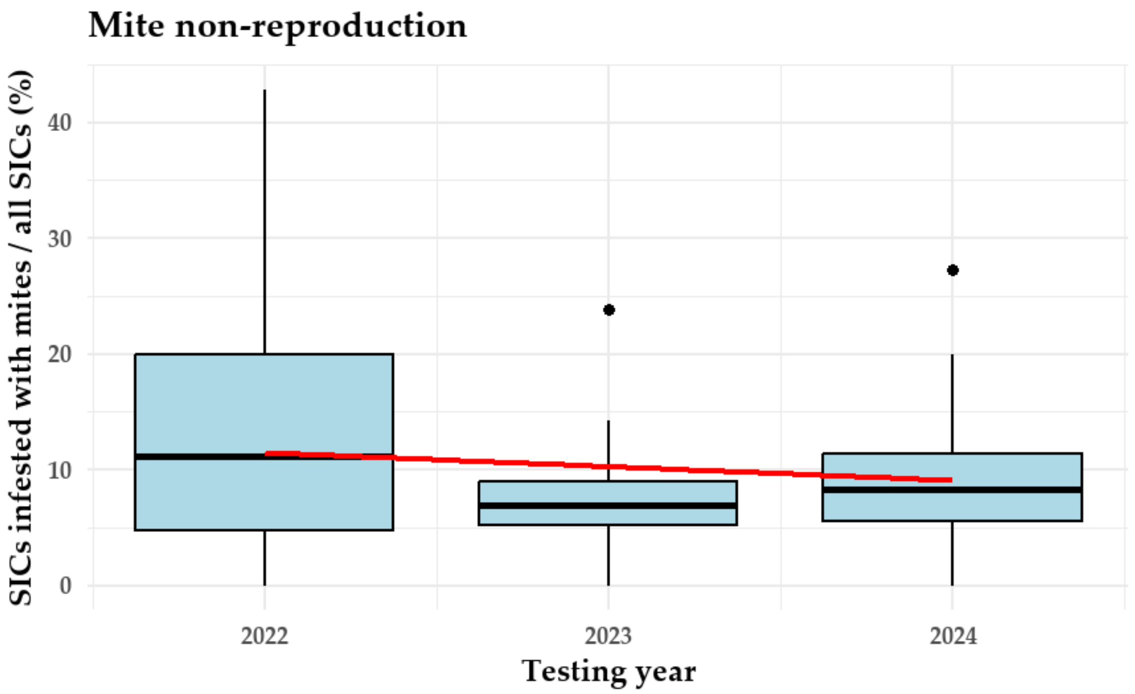

SICs = Single Infested Cells

Supplementary Figure S10. Boxplots of the mean Varroa reproduction rate scores per testing year (2022-2024).

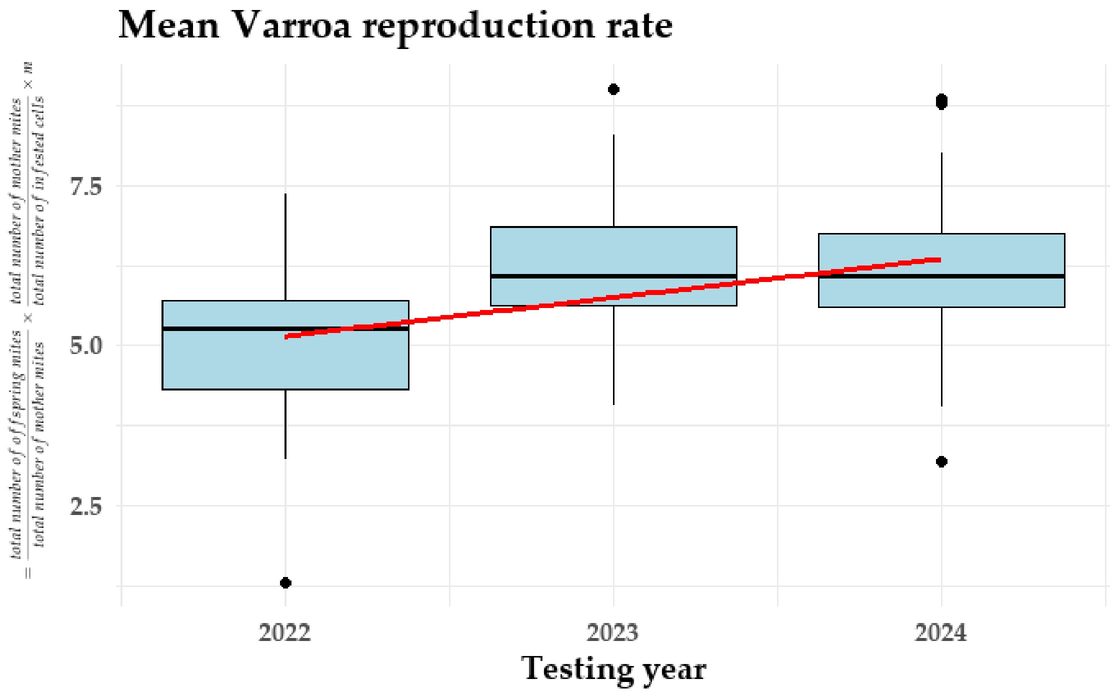

Supplementary Table S1. Representativeness of the data collection over testing years.

**Testing year**	**2017**	**2018**	**2019**	**2020**	**2021**	**2022**	**2023**	**2024**
Number of beekeepers	25	20	27	32	49	42	42	45
Number of tested queens	182	173	205	261	374	264	284	278
Mean number of tested queens per beekeeper	7.28	8.65	7.59	8.16	7.63	6.29	6.76	6.18
Mean number of traits tested per queen	7.75	7.33	7.02	6.44	5.69	6.13	4.48	4.60
Percentage of queens tested for all 3 behavior traits	95.05	89.02	73.17	70.11	67.11	70.45	65.49	44.60
Percentage of queens tested for all 2 productivity traits	51.65	36.42	30.24	28.35	37.97	42.80	23.59	30.22
Percentage of queens tested for all 4 resilience traits	6.59	10.40	20.49	26.05	0.00	11.74	8.10	16.55
Percentage of queens tested for the SOV trait	80.22	31.79	69.27	59.39	76.47	84.09	73.59	63.31
Number of top queens	38	40	58	43	63	61	41	39
Number of top queens with tested offspring	8	11	13	11	11	13	10	-
